# Why Are We Right-Handed

**Published:** 1896-06

**Authors:** 


ARTICLE VI.

### WHY ARE WE RIGHT-HANDED.

In all communities left-handed individuals seem to occur in somewhat varying proportions. Among ourselves about one in fifty is said to be left-handed. There is no doubt, from frequent experience, that the peculiarity is hereditary, so that we would not be much surprised if a race were met in which left-handedness was the rule and not the exception. Yet the reversal of so general a law as that of prevalent right-handedness would need to be established by very conclusive evidence, and the statements have been made as to a preponderance of left-handed individuals in various parts of the world, none of them are supported by such careful and prolonged observation of facts as would be necessary for their unhesitating acceptance.

One of the prevailing ideas about right-handedness is that it is merely a matter of training, and that left-handed individuals have become so either from the want of care on the part of nurses and parents or from imitation of some older person. In many children the preference for one hand is shown from a very early age, before the child has learned to handle anything but the very simplest toys, and, therefore, before training can have caused a preference at all. More than this, the experience of left-handed persons is on record in whom the peculiarity has been early noticed and combated, but without the slightest effect.

It is well known that, though our external configuration is so nearly symmetrical, the arrangement of the internal organs is very different. The heart lies obliquely in the chest, and more to the left side than to the right; the liver, by far the heaviest of the internal organs, is on the right side; the two lungs are differently shaped; and moreover, the blood-vessels supplying the two sides, especially in the upper regions of the body, are differently disposed. It is natural that these irregularities of arrangement should have been thought, in some way or other, to supply the explanation.

There is, however, one extremely curious and interesting instance of want of symmetery in the bodily functions which is not merely analogous to right handedness, but closely linked with it. The nervous machinery normally connected with speech is situated on one side of the brain only. So intimate is the relation of this subject to right-handedness that we must consider it in some detail.

It is well known that each side of the brain is connected with the movements and sensations mainly on the opposite side of the body; the right brain moves the left arm and leg, and vice versa. Now, cases are not infrequent in which, with or without "a shock," or at least some degree of obvious loss of muscular power on the right side of the body, the faculty of recalling and reproducing spoken words is totally, or almost totally, lost. Such loss of speech is technically called aphasia. It was first shown some thirty-five years ago by a French physician that this particular symptom is associated with damage to a limited and very definite part of the brain substance on the left side, which has since been known, in honor of its discoverer, as Broca's convolution. When the power of speech has thus been lost it is possible, if the mental faculties are not otherwise damaged, to acquire it again by just such a course of training and practice as the child passes through in learning to speak at first, even where Broca's convolution has been so damaged as to be quite incapable of performing its functions. In such a case the portion of the brain on the right side corresponding to Broca's convolution is capable of taking up its work, but only by being educated to do so, just as the damaged portion of the brain had been originally. If after this the power of speech is lost again by damage to the right side similar to that which impaired the left, there is no hope of its being restored a second time.

It is thus clear that there are two organs or portions of the brain capable of controling speech, and that under ordinary circumstances only one of them is trained to do so, the other lying fallow. All the education is given to one favored side, and all the work is done by it; but the neglected one, if called by necessity to undertake the work, can be trained to do it, and to do it, apparently, as satisfactorily as the other.

Here, then, is a singularly complete analogy to the preferential use of the right hand: There are two sets of organs, either of which may be used for speech, one on each side of the brain, but only those upon one side are trained; only they have the education carried out which makes them effective. Yet if the educated centers are so damaged as to lose their functions, the others can be trained to take their place. So we have two hands, either of which may be trained for the performance of delicate movements; yet in most of us only one of them has been so trained; the other remains comparatively awkward and inactive, unless accident compels it to try to take the place of the educated hand.

A striking analogy; but it is more than an analogy. We have said that the active speech center is that on the left side, and this is the case in the great majority of individuals. But occasionally it is found that the right and not the left side of the brain has been educated as regards speech. When this is the case, it is always found that the individual has been left-handed. Whatever, then, is the cause of right-handedness, it is closely associated with left-brainedness, if we may use the expression, not only for the comparatively coarse movements of the hand, but for the fine adjustments of windpipe, tongue, lips, etc., which produce articulate speech, and the far finer machinery within the brain itself, which registers our stores of words.

When a child displays a decided preference for the use of the left hand it is, as we have seen, useless to make forcible efforts to suppress it. By all means let the right hand be trained in writing, in using knife and spoon at table and in as many actions usually right-minded as can be easily superintended. But the use of the left on other occasions should not be prevented; this will only diminish its training and its aptitude, without greatly increasing the dexterity of the right.

There seems to be no good reason why right-handed people should not attain some of the ambidexterity which is usually the privilege only of the left-handed. A little trouble expended in practicing with the left hand as well as the right, throwing, drawing and other common movements requiring skill, would be rewarded by a much increased usefulness of that generally neglected member. If there is a natural preference for the right hand, it is probable that no amount of practice would make the left equally expert in actions that have once been well acquired by the right. But the experience of the left handed seems to show that it is well worth while for the right handed to give more attention to their despised left hands than they usually do—*Chambers' Journal.*

